# Mechanisms of energy metabolism reprogramming and homeostasis maintenance in overwintering hibernating animals

**DOI:** 10.3389/fvets.2026.1818015

**Published:** 2026-06-11

**Authors:** Ye Tian, Guangyu Jiang, Tingting Wang, Xinyu Dong, Qing Liu, Guanzhong Hu, Hang Qi, Huimei Yu

**Affiliations:** 1 Department of Pathophysiology, College of Basic Medical Sciences, Jilin University, Changchun, Jilin, China; 2 Department of Gynecological Oncology, Jilin Provincial Cancer Hospital, Changchun, Jilin, China

**Keywords:** hibernation, hypothermia, lipid metabolism, oxidative stress, torpor

## Abstract

Hibernation is a specialized adaptive energy-saving survival strategy evolved by animals to withstand winter cold stress and food scarcity. Its core feature lies in profound metabolic suppression, characterized by a drastic reduction in metabolic rate during hibernation, accompanied by the coordinated downregulation of multiple physiological functions such as body temperature, heart rate, and respiratory rate. The establishment and maintenance of this deep metabolic suppression state essentially rely on the systemic reprogramming of energy metabolism, which serves as the core driving force of hibernation adaptation. During this reprogramming process, lipid metabolism acts as a key executive link: fats stored in adipose tissue not only function as the primary energy reserve pool during hibernation but also undergo precise regulatory remodeling in terms of their compositional characteristics, mobilization efficiency, and catabolic processes, thereby synchronously adapting to the demands of energy supply and environmental adaptation goals. Importantly, metabolic suppression often precedes cooling and can exceed Q_10_ predictions, indicating active regulatory control rather than passive thermal effects. Reliance on lipid oxidation and cyclic torpor–arousal transitions should heighten oxidative stress risk: electron leakage from mitochondrial complexes I/III during deep torpor, relative hypoxia from reduced perfusion, and rapid “metabolic restart” upon arousal may resemble ischemia–reperfusion. Yet hibernators show minimal oxidative damage, implying robust antioxidant and repair programs. This review summarizes recent advances in the metabolic remodeling of lipids, substrate conversion, and oxidative stress adaptation in hibernating animals. It reveals the evolutionary mechanisms underlying energy metabolism adaptation and provides potential insights for applications in metabolic diseases, cryobiology, and related fields.

## 1 Introduction

Winter poses severe challenges of low temperature and food scarcity for many animals, and hibernation has evolved as a sophisticated adaptive strategy to overcome these adversities. During hibernation, animals undergo profound physiological transformations—including a drastic reduction in metabolic rate, significant drops in body temperature, heart rate, and respiratory rate, as well as shifts in energy utilization patterns—all collectively designed to minimize energy expenditure and sustain survival for months without continuous feeding (1, 2).

Among these adaptive changes, lipid metabolism is regarded as the “core chassis” for hibernating animals to maintain energy homeostasis and tissue function during overwintering. On the one hand, adipose tissue undergoes rapid proliferation, differentiation, and lipid droplet remodeling prior to hibernation, establishing a sustainably mobilizable energy reservoir for the hibernation period; brown adipose tissue (BAT), a thermogenic organ unique to mammals, maintains the animals’ hypothermic homeostasis through non-shivering thermogenesis (NST) in low-temperature environments (3–5). On the other hand, significant shifts in energy substrate utilization occur during hibernation: glucose oxidation is downregulated, and fatty acid β-oxidation becomes the primary source of ATP, while concurrently producing metabolic water to alleviate dehydration stress induced by prolonged fasting. Further refined adaptations are reflected in the “division of labor and collaboration” at the level of fatty acid composition, with overwintering animals exhibiting a strategy of selective mobilization and retention of fatty acids. Saturated fatty acids (SFAs) tend to support long-term energy supply, while polyunsaturated fatty acids (PUFAs) maintain the low-temperature fluidity of cell membranes and lipid depots by reducing the melting point of membrane lipids, ensuring that fats remain mobilizable at low temperatures; however, the double-bond structure of PUFAs also renders them more susceptible to lipid peroxidation, creating a trade-off between “demands for low-temperature fluidity” and “burden of oxidative stress” (6).

The traditional view once attributed hibernatory hypometabolism primarily to reduced enzymatic reaction rates induced by low temperatures. Nevertheless, recent experimental and comparative physiological studies have demonstrated that metabolic suppression often precedes a decrease in body temperature, and in some species, a metabolic reduction far exceeding Q₁₀-predicted levels can occur even with limited decreases in body temperature. This suggests that actively regulated metabolic suppression mechanisms dominate mammalian hibernation, rather than passive thermal effects alone (7).

While this reliance on fatty acids sustains the entire torpor–arousal cycle, it also creates distinct windows of oxidative stress. During deep torpor, profound reductions in body temperature, heart rate, blood flow, and mitochondrial respiration can facilitate electron leakage from the mitochondrial respiratory chain (especially complexes I and III), allowing electrons to react with oxygen and continuously generate reactive oxygen species (ROS) (8). Chronic hypoperfusion and relative hypoxia further disrupt cellular redox homeostasis, potentially sensitizing high-energy-demanding organs such as the heart and brain. In contrast, periodic arousal represents a rapid metabolic “reboot” in which oxygen consumption and mitochondrial respiration surge within hours, theoretically triggering an oxidative burst. Reperfusion of previously underperfused tissues may also induce ischemia–reperfusion-like injury. Strikingly, however, many hibernators exhibit minimal evidence of oxidative damage across the hibernation season, suggesting the evolution of potent, tightly regulated antioxidant and repair systems (9). A growing body of evidence suggests that redox sensors upregulate the expression of antioxidant enzymes via ARE-dependent transcription (10). Meanwhile, lipid metabolism promotes the expression of fusion-related proteins (e.g., Mfn1/2, OPA1) by activating the PPAR*α*/PGC1-α signaling pathway, thereby protecting mitochondrial integrity (11). This review summarizes the major sources of oxidative stress during hibernation and the coordinated molecular programs that preserve redox homeostasis and mitochondrial structure.

## 2 Hibernating animals respond to low temperatures by adjusting fat and fatty acid composition

### 2.1 Distinct roles of different adipose tissue types during hibernation

Adipose tissue in mammals primarily exists in three specialized forms: white adipose tissue (WAT), BAT, and beige adipose tissue (12). Strictly seasonal hibernators enhance adipogenesis prior to hibernation, thereby enabling the rapid synthesis and accumulation of sufficient lipids in WAT, which serves as the primary “fuel” source to sustain winter survival (13). Species specialized in fat storage generally experience a pronounced phase of hyperphagia over several weeks to months prior to hibernation, thereby accumulating substantial internal fat reserves. A typical case is the golden-mantled ground squirrel (*Callospermophilus lateralis*), which exhibits a twofold increase in body mass and a threefold rise in fat mass within a mere 5–7 weeks before entering hibernation (14). Meantime, WAT undergoes a pre-hibernation remodeling process. During the hibernation preparation period of Syrian hamsters, adipocytes in the inguinal white adipose tissue (iWAT) are fragmented into smaller lipid droplets compared to their normal state, facilitating lipid catabolism for energy provision during hibernation (15). Such histological morphological changes can reflect variations in the mobilization or accumulation of stored lipids.

Functionally, WAT serves as the primary energy storage organ in mammals, whereas BAT functions predominantly in energy dissipation for thermogenesis (16). BAT is a unique thermogenic organ exclusive to mammals (17, 18). In small mammals (e.g., rodents) and human infants, cold stimuli induce the activation of BAT, and its thermogenic capacity is primarily mediated by NST (16). This process relies on the uncoupling effect driven by uncoupling protein 1 (UCP1), a mitochondrial protein (19). UCP1 has been identified as the sole essential protein for BAT-mediated thermogenesis and lacks functional expression in other tissues, thus serving as a specific marker for adipocyte thermogenesis (18, 20). By uncoupling the mitochondrial respiratory chain from oxidative phosphorylation, UCP1 dissipates energy as heat, a function critical for organisms to cope with cold stress (19).

In addition to WAT and BAT, beige adipose tissue was later identified as a distinct type of adipose tissue. It is composed of “beige adipocytes” that reside within white adipose tissue. These cells exhibit morphological and functional similarities to brown adipocytes, characterized by the presence of multilocular lipid droplets and abundant UCP1-positive mitochondria, and are therefore also referred to as “inducible brown adipose tissue”. At the molecular level, beige adipocytes co-express brown adipose tissue-specific genes, including UCP1, PGC-1α, and PRDM16 (15, 17, 21). Peroxisome proliferator-activated receptor *γ* (PPARγ), the core regulator of adipose browning, participates in the differentiation of both white and brown adipocytes. Its full agonist can promote PRDM16 accumulation by stabilizing the ligand-binding domain, thereby inducing the expression of browning-related genes such as UCP1 and CIDEA (17, 22–24). Among various white fat depots, the iWAT serves as a major site for beige adipose tissue enrichment, as it readily forms thermogenically active beige-like cells under physiological stimuli such as cold exposure (25).

### 2.2 Adipose tissue and skeletal muscle serve as central regulators of hibernation energy metabolism

WAT functions as the primary energy reservoir with temporally regulated substrate release, BAT governs thermoregulation and rapid temperature recovery, and skeletal muscle serves as both an energy buffer and a metabolic regulator (26–28). Through dynamic coordination and reciprocal signaling, these three tissues collectively drive adaptive metabolic remodeling, maintain whole-body energy homeostasis, and ensure survival and rapid functional recovery of hibernators throughout the overwintering period.

During the pre-hibernation fattening phase, WAT serves as the primary energy reservoir, accumulating large amounts of triglycerides to establish sufficient lipid stores for the subsequent prolonged hibernation (26). Concurrently, BAT enhances thermogenic capacity, and skeletal muscle adjusts substrate utilization and metabolic flexibility, collectively preparing the organism for the drastic metabolic shifts that occur during torpor (27).

In deep torpor, the body maintains a hypothermic and hypometabolic state. Lipolysis in WAT is moderately restrained to balance energy supply and prevent excessive fatty acid release; BAT sustains low-level basal non-shivering thermogenesis to stabilize core temperature; skeletal muscle sharply reduces energy consumption, relies predominantly on fatty acids and ketone bodies, and minimizes disuse atrophy during extended inactivity (28).

During periodic arousals, metabolic activity rebounds rapidly. WAT undergoes robust lipolysis to release abundant free fatty acids, which fuel intensive BAT-mediated thermogenesis for rapid body temperature recovery and simultaneously provide energy substrates for skeletal muscle. Skeletal muscle further contributes gluconeogenic precursors to support systemic glucose homeostasis and modulates whole-body lipid distribution and energy flux (2).

### 2.3 Selective mobilization of fatty acids

Fatty acids are classified into different categories according to two main criteria: carbon chain length (short-chain, medium-chain and long-chain fatty acids) and degree of unsaturation (saturated, monounsaturated and polyunsaturated fatty acids). The former classification is based on the number of carbon atoms (e.g., short-chain fatty acids: fewer than 6 carbons, medium-chain fatty acids: 6–12 carbons, long-chain fatty acids: more than 12 carbons), whereas the latter is defined by the existence and quantity of carbon–carbon double bonds. The diverse physiological functions of fatty acids collectively allow hibernating animals to efficiently mobilize and utilize stored fat reserves, thus maintaining vital physiological functions under long-term extreme environmental conditions (29).

#### 2.3.1 Long-chain fatty acids: core energy substrates during fasting periods

Metabolic adaptation is the core survival strategy for all organisms to cope with energy deficiency, allowing their survival under starvation conditions such as fasting (30). For mammals, the “core mechanism” of this metabolic adaptation encompasses two key components: utilizing long-chain fatty acids (LCFAs) as the primary energy source while reserving glucose for preferential supply to critical organs (e.g., the brain) that cannot efficiently utilize LCFAs. Glycogen stores in the body can sustain energy supply for less than 1 day, whereas LCFAs released from the hydrolysis of triglycerides stored in adipose tissue can meet energy demands for several months—directly confirming that LCFAs serve as the “material basis” for core energy substrates during fasting (30, 31).

Within the classification system based on carbon chain length, LCFAs exhibit a significantly higher energy density per unit mass than other fatty acid classes (32, 33). At physiological pH, LCFAs are amphipathic molecules that are insoluble in aqueous environments due to their hydrophobicity, necessitating emulsification by bile salts to facilitate their digestion and absorption (34, 35). This structural property dictates that LCFAs are not readily catabolized and utilized *in vivo*; instead, they are stored in the form of fat to serve as reserve energy sources during hibernation. Furthermore, their hydrophilic carboxyl groups bind to plasma albumin, thereby enabling their transport in the bloodstream (36, 37).

LCFAs are absorbed via two distinct mechanisms (38). Skin epithelial cells and similar tissues require baseline levels of LCFA for fundamental membrane structure synthesis, thus primarily absorbing LCFA via simple diffusion mechanisms. Previous studies have indicated that skin LCFAs indirectly modulate systemic energy metabolism through their physical barrier function, rather than directly participating in β-oxidation (32). In contrast, LCFAs serve as the primary energy source for cardiomyocytes, providing approximately 60–90% of the ATP required for sustained cardiac contraction (39, 40). Cardiomyocytes, which rely on an efficient and stable supply of LCFAs to sustain their physiological functions, primarily adopt a protein-mediated uptake mechanism dominated by fatty acid transport proteins (FATPs) to maintain energy homeostasis (37, 41, 42). Regarding this uptake pathway, there remains ambiguity as to whether FATPs function as “true transporters” or indirectly promote intracellular LCFA accumulation by inhibiting their efflux (43).

#### 2.3.2 PUFAs as key determinants of membrane fluidity and fat mobilization at low temperature

PUFAs are regarded as the core factor for maintaining the fluidity of membrane phospholipids and adipose tissue under low-temperature conditions. As the main structural component of membrane phospholipids, fatty acids directly determine the fluidity of cell membranes: when ambient temperature decreases, regular phospholipid bilayers transition from a fluid “liquid-crystalline phase” to a rigid “gel phase.” This reduction in fluidity leads to increased membrane thickness, which in turn impairs its normal function (44–46). However, such phase transition has not been observed in the cell membranes of hibernating animals (45). Existing studies have confirmed that the increased proportion of PUFAs in membrane phospholipids is the key mechanism to inhibit this phase transition—desaturases introduce double bonds into fatty acids, and these double bonds, especially cis double bonds, can loosen the packing of the membrane bilayer and enhance fluidity, thereby ensuring the biological function of cell membranes after temperature decline (47, 48).

At the adipose tissue level, fat depots of homeothermic animals solidify at 20–32 °C, while PUFA-enriched fat depots in hibernators such as ground squirrels remain liquid even at −2 °C. Given that solidified fats cannot be efficiently mobilized and utilized as metabolic substrates, maintaining the fluidity of adipose tissue serves as a fundamental prerequisite for fat catabolism and energy metabolism during hibernation (45, 49). Obligate homeothermic mammals strictly maintain their core body temperature within a stable warm range far above the fat solidification threshold, so they are rarely confronted with the risk of fat solidification. In contrast, hibernating species are able to enter torpor and survive at ambient temperatures well below 20 °C. Even with extremely low dietary intake of PUFAs, these animals can sustain adipose tissue fluidity via endogenous physiological regulatory pathways, rather than relying on sufficient exogenous PUFA supply. This adaptive mechanism ensures sustained fat mobilization and stable energy provision, which further supports essential physiological activities throughout long-term low-temperature hibernation (50).

Mammals are unable to synthesize PUFAs *de novo* due to the lack of key enzyme systems, so their PUFA requirements are entirely dependent on dietary intake (51, 52). For hibernating mammals, they significantly increase the relative content of PUFAs in adipose tissue through active feeding before torpor. Previous studies have shown that the high-PUFA diet can also prolong torpor duration, reduce metabolic rate, and enhance the hibernation ability of hibernating animals in low-temperature environments (53–56). Experimental evidence has demonstrated that the subtype ratio of polyunsaturated fatty acids does not affect the duration and depth of hibernation in hibernating animals (57).

A three-year field study first confirmed that the dietary PUFA level of wild hibernating animals can significantly affect their subsequent hibernation phenotype (54). By modulating dietary components, particularly fatty acid composition, the torpor depth and duration of hibernating animals can be artificially regulated. More importantly, the study identified an optimal dietary PUFA concentration range of 33–74 mg/g: within this range, it not only meets the physiological needs of cell membrane fluidity during hibernation but also limits oxidative stress to a tolerable level for the organism, thereby ensuring hibernation efficiency (55, 58). Once PUFA intake exceeds this threshold, it exacerbates lipid peroxidation, and the toxic lipid peroxides produced damage hibernation-related metabolic organs such as the intestine and adipose tissue (54). Eventually, this forces animals to shorten the hibernation cycle and arouse early to initiate normothermic metabolic repair mechanisms, leading to a significant decline in hibernation quality.

#### 2.3.3 Saturated fatty acids also serve as a source of sustained stable energy

SFAs, characterized by exclusively single carbon–carbon bonds and fully hydrogenated hydrocarbon chains, exhibit a tightly packed molecular structure that confers a high energy density per unit mass (59, 60). This stable molecular architecture results in a relatively slow metabolic rate *in vivo*, as their saturated bonds are less susceptible to enzymatic breakdown. Consequently, the gradual catabolism of SFAs enables a sustained energy supply over extended periods, a critical trait for organisms relying on long-term energy reserves, such as hibernating animals during dormancy.

Due to their higher calorific values (kcal/g) compared to unsaturated fatty acids, saturated fatty acids (SFAs) can serve as efficient energy substrates in hibernating animals under certain conditions (61). Some studies have reported that specific hibernators preferentially catabolize SFAs during torpor, while conserving other fatty acid types in storage to optimize energy utilization (62, 63). However, the patterns of fatty acid mobilization are highly species-specific and can be influenced by torpor depth, diet, and tissue-specific lipid composition. For instance, under normal physiological conditions, fatty acids with shorter chain lengths and higher degrees of unsaturation are generally more readily mobilized than other types (64). Therefore, although SFAs may be an important energy source in some hibernators, their preferential utilization should not be generalized across all species or tissues.

Consistent with the species- and tissue-specific nature of fatty acid utilization, fatty acid profiles in adipocytes from summer-active versus hibernating squirrels revealed that specific SFAs, such as palmitic acid (16:0) and stearic acid (18,0), were mobilized at higher rates relative to the average, whereas certain unsaturated fatty acids, particularly oleic acid (18,1ω9) and linoleic acid (18,2ω6), were preferentially retained (65). These observations indicate that, at least in the adipose tissue of hibernating squirrels, selective mobilization mechanisms favor specific SFAs, highlighting the nuanced and species-dependent nature of lipid utilization during hibernation.

## 3 Fatty acid metabolism serves as the primary energy supply mechanism under low-temperature conditions

### 3.1 Low metabolic rate in animals during hibernation

Metabolic suppression stands as the core hallmark of hibernation (45). During hibernation, the overall metabolic rate of hibernating animals drops to 5–30% of their basal metabolic rate (2). In the state of deep torpor, the basal metabolic rate of hibernators can be further diminished to 2–4% of that in the active phase; under extreme conditions, the metabolic rate of some species may even drop to 1%. Concomitant with this metabolic suppression is a marked downregulation of a suite of physiological functions (66). During this period, the minimum body temperature can decrease to 2.9 °C; the heart rate decreases from the normal range of 200–300 beats per minute to 3–5 beats per minute, while the respiratory rate slows from 100 to 200 breaths per minute to 4–6 breaths per minute. Some small rodent species exhibit intermittent respiration, characterized by several deep breaths followed by a pause lasting minutes to tens of minutes (8, 67, 68). Furthermore, these physiological parameters can rapidly recover to levels close to normal during periodic arousals.

#### 3.1.1 Low metabolic rate is not merely a consequence of passive thermal effects

Temperature reduction is generally recognized to slow the rate of enzyme-catalyzed reactions via the “Q₁₀ effect.” According to this principle, a 10 °C decrease in temperature reduces the rate of chemical processes by 2–3 times. Simultaneously, the “neutral pH” of intracellular fluids increases as temperature drops (69). However, the “optimal pH” for enzymes in animals has evolved over long periods to suit warm physiological environments and cannot adjust synchronously with sudden drops in body temperature (Tb). This pH shift also inhibits enzymatic reaction rates.

However, multiple lines of evidence indicate that passive thermal effects are not the core driver of metabolic suppression. During hibernation, the core Tb of black bears (*Ursus americanus*) drops only 5–6 °C, yet their metabolic rate decreases by 75%, significantly exceeding the range predicted by the Q_10_ effect (1); The edible dormouse(*Glis glis*) can enter hibernation even at 28.6 °C (thermoneutral temperature) (70). Furthermore, the reduction in metabolic rate often precedes the drop in Tb during torpor entry, suggesting that an active, regulated metabolic suppression mechanism plays a dominant role in mammalian hibernation (71).

#### 3.1.2 Specific neural activity can induce a hibernation-like state

Several studies have shown specific neural activities can induce hibernation-like physiological changes even in the absence of external energy restriction (72). In mice, activation of specific neurons within the hypothalamic preoptic area can rapidly decrease core body temperature to 20 °C and depress metabolic rate, thereby mimicking natural hibernation patterns. These neurons are highly active during fasting-induced torpor, and silencing them blocks the progression of hibernation. Previous studies have found that a population of Qrfp gene-expressing neurons (hereafter referred to as Q neurons) exists in the anteroventral periventricular nucleus (AVPe) and medial preoptic area (MPA) of the hypothalamus; activation of these neurons can induce rodents to enter a hibernation-like state that persists for more than 48 h (73, 74).

#### 3.1.3 Reversible inhibition of mitochondrial respiration

Reversible inhibition of mitochondrial respiration is a putative mechanism for metabolic suppression during hibernation (75, 76). Crucially, this inhibition exerts a more decisive effect on the reduction of overall metabolic rate during the initial phase of torpor prior to a marked drop in body temperature (Tb). This view is further supported by research conducted on thirteen-lined ground squirrels (*Ictidomys tridecemlineatus*), which has uncovered distinct tissue-specific variations in mitochondrial respiratory capacity across hibernation periods. Compared with the summer active state, hepatic mitochondrial respiration decreased by 70%. Moderate suppression was observed in mitochondria of skeletal and cardiac muscles, whereas no obvious inhibitory effect was detected in cerebral cortex mitochondria. Mitochondrial respiration suppression is not attributed to impaired substrate transport or metabolite accumulation, but is predominantly regulated via active post-translational modifications (8). Among these regulatory pathways, the soluble adenylate cyclase (sAC)-protein kinase A (PKA) pathway rapidly suppresses mitochondrial respiration via phosphorylation (77). In parallel, SIRT3-mediated acetylation contributes to the regulatory process by modulating the activity of electron transport system (ETS) complex II (78, 79). Both modifications are regulated by temperature-sensitive enzyme activity, collectively achieving profound and reversible inhibition of mitochondrial respiration during hibernation.

### 3.2 The shift in substrate utilization for energy metabolism during animal hibernation

#### 3.2.1 Fatty acids as a superior energy source over glucose

During hibernation, carbohydrate oxidation significantly decreases, and hibernating organisms rely primarily on fat burning as their main source of metabolic energy (62, 80). In the field of animal physiology, the respiratory quotient (RQ) serves as a crucial indicator for measuring the substrate type in an organism’s energy metabolism, accurately reflecting the consumption ratio of different energy-yielding substances during metabolic processes. A respiratory quotient of 1.0 indicates carbohydrates as the energy source, while a value of 0.7 signifies lipids as the primary energy source (81). Hibernating animals typically exhibit respiratory quotients close to 0.7 during dormancy, confirming fatty acids as the predominant energy source at this stage (45). Multiple studies have validated this shift in metabolic pathways by measuring parameters including glycolytic rates, glycolytic enzyme activity, the metabolic fates of radioactively labeled glucose as a carbon source, lipogenesis and lipolytic enzyme activity, and the gluconeogenic capacity using different metabolic substrates (45, 82, 83). These studies have concluded that although hibernating animals may exhibit greater anaerobic glycolytic capacity than non-hibernators, less glucose is oxidized during the hibernation period.

The key advantage of fatty acids serving as an energy source during hibernation resides in their substantially higher energy density relative to glucose (84). Each gram of fatty acids contains 9.3 kcal of energy, whereas glucose provides only 4.0 kcal/g. This means that each gram of fatty acids releases more than twice the energy of an equivalent mass of carbohydrates (85). In terms of metabolic efficiency, fatty acids yield 2.6 times more electrons than glucose, directly resulting in a 2.5-fold increase in ATP production efficiency. Taking palmitic acid as an example, one molecule of palmitic acid undergoing β-oxidation yields 106 ATP, whereas one molecule of glucose undergoing glycolysis produces only 30–32 ATP (86). More significantly, the storage and transport of fatty acids in organisms do not require association with water. Additionally, the water produced during fatty acid metabolism far exceeds the water consumed, reducing the organism’s dependence on external water sources while providing energy (87). The net water production from fatty acid metabolism can reach four times that of glucose metabolism. This characteristic enables hibernating animals to synchronize energy supply with water maintenance through lipid metabolism, effectively alleviating dehydration stress during months of fasting.

#### 3.2.2 Relevant signaling pathways in the shift of metabolic substrates

##### 3.2.2.1 The PPAR*α*/PGC-1α axis: a dominant regulator orchestrating metabolic substrate switching in physiological adaptation

A hallmark metabolic shift in hibernating animals is the transition from a carbohydrate-dominated to a lipid-dominated metabolic profile, a process orchestrated by the PPAR*α*/PGC-1α axis (88, 89).

Peroxisome proliferator-activated receptor α (PPARα) emerges as a pivotal nuclear receptor orchestrating systemic energy metabolism, with predominant expression in metabolically active tissues, including the liver, skeletal muscle, BAT, and kidneys (90, 91). Its primary biological role centers on unlocking stored lipids for energy production, a function that becomes particularly prominent under metabolic stressors such as fasting, physical exertion, or elevated energy demand (92). This regulatory axis exhibits striking evolutionary conservation across vertebrates: in hibernators like the thirteen-lined ground squirrel (*Spermophilus tridecemlineatus*), PPARα is robustly activated by the combined cues of hypothermia and prolonged fasting during torpor (28). This activation triggers two interconnected metabolic cascades: enhanced lipolysis in WAT to release free fatty acids, and concurrent induction of hepatic fatty acid oxidation and ketogenesis—ensuring a steady supply of alternative energy substrates (fatty acids and ketone bodies) to sustain vital organs like the brain and heart when glucose availability is limited. In humans, a comparable response is observed following 12 h of fasting, where hepatic PPARα activation upregulates lipolytic gene expression, elevates plasma free fatty acid concentrations, and shifts the primary energy source from carbohydrates to lipids (93).

To exert its transcriptional regulatory effects, PPARα forms a functional complex with PGC-1α, which then binds specifically to PPAR response elements (PPREs) in the promoter regions of target genes (94). This molecular interaction orchestrates a coordinated metabolic program: it first upregulates the expression of lipolysis-related genes, including adipose triglyceride lipase (ATGL) and hormone-sensitive lipase (HSL), to promote lipid mobilization (95–97). Concomitantly, PPARα synchronously activates the fatty acid *β*-oxidation pathway by inducing the expression of mitochondrial fatty acid transport genes such as carnitine palmitoyltransferase 1 (CPT1) (98). This sequential regulation accelerates the breakdown of free fatty acids into acetyl-coenzyme A (acetyl-CoA), which is subsequently funneled into the tricarboxylic acid cycle for efficient energy production.

PPARα serves as a central transcriptional switch for long-term metabolic adaptation to cold in animals. Recent studies have clearly demonstrated that the role of PPARα in BAT thermogenesis exhibits temporal specificity. Under short-term cold stimulation, PPARα is not activated to regulate thermogenesis; however, under long-term cold adaptation conditions, PPARα is specifically activated, thereby promoting NST to maintain body temperature. Studies have found that chronic cold exposure drives the expression of PPARα by activating Pparα-En4, a brown adipose tissue-specific enhancer; inhibition of this enhancer impairs the long-term cold tolerance of mice. This finding further corroborates the critical role of PPARα (99).

##### 3.2.2.2 PDK: suppressing carbohydrate oxidation via PDH inhibition

To investigate the fuel utilization switching mechanism during hibernation in the 13-lined ground squirrel (*Spermophilus tridecemlineatus*), researchers focused on the differential expression of pyruvate dehydrogenase kinase 4 (PDK4) while analyzing its hibernation-upregulated genes (100). Under the low-temperature environment of hibernation, the nuclear receptor PPARα is specifically activated, and the activated PPARα binds to the response elements in the PDK4 gene promoter to substantially induce PDK4 upregulation. Subsequently, PDK4 specifically inhibits the activity of pyruvate dehydrogenase (PDH), blocking the entry of glycolytic products into the tricarboxylic acid cycle and thereby minimizing carbohydrate oxidation (101–103). Compared to the active summer period, PDK4 protein expression levels were significantly elevated in the heart, skeletal muscle, and white adipose tissue of ground squirrels during hibernation (104).

Studies have shown that changes in PDK4 expression are closely associated with dynamic fluctuations in hormone levels. Researchers found that injecting exogenous insulin into diabetic rats can significantly reduce the levels of PDK4 protein and mRNA in cardiac and skeletal muscle tissues (105, 106). During the torpor stage of hibernation, the metabolic activity of pancreatic β-cells in ground squirrels decreases under the combined effects of low-temperature stress and interrupted glucose sources (e.g., dietary carbohydrates), directly reducing insulin synthesis and secretion. This decline in insulin levels further promotes PDK4 gene transcription and expression (104).

In the early stage of hibernation, serum insulin levels remain relatively high, primarily due to the overall suppression of systemic metabolic rate and the development of insulin resistance (107). At this stage, the activation of PPARα by free fatty acids not only enhances fatty acid β-oxidation but also initiates PDK4 gene expression, thereby inhibiting carbohydrate oxidation (100, 105).

Additionally, other studies have shown that insulin levels in the pancreatic tissue of hibernating animals exhibit a steady upward trend prior to spring arousal. This change is hypothesized to promote the formation of a “rapid insulin release reserve pool” in the pancreas, ensuring the immediate secretion of insulin upon arousal to meet the body’s demand for rapid insulin uptake during the resumption of feeding (108).

##### 3.2.2.3 PTL: mediating cold-adapted lipolysis via hormone-independent low-temperature activity

Pancreatic triacylglycerol lipase (PTL) serves as another key protein enabling hibernating mammals to shift their metabolic substrate reliance toward fats. Under normal physiological conditions, PTL is a pancreas-specific enzyme, exclusively expressed in the pancreas, which hydrolyzes the ester bonds in triacylglycerols and participates in lipid catabolism. However, studies have demonstrated that during hibernation, the levels of PTL protein and mRNA are significantly upregulated in non-pancreatic tissues including the heart and WAT (109).

More importantly, PTL possesses distinct functional advantages: it is hormone-independent and exhibits extremely low temperature sensitivity, retaining activity even at temperatures as low as 0 °C, which perfectly fulfills the requirement for cold-adapted lipolysis during hibernation. This characteristic enables it to function complementarily with HSL. Since HSL activity is regulated by hormones such as insulin and catecholamines, fluctuations in hormone levels during hibernation may lead to its functional instability (109). PTL’s non-hormone-dependent nature and low-temperature activity precisely compensate for this potential disruption. Together, they form a “dual lipolysis system” that collectively ensures the continuous progression of fat breakdown during hibernation (45, 110).

#### 3.2.3 Ketone bodies: key energy substrates and metabolic regulatory signaling molecules during hibernation

Under fasting conditions, when acetyl-CoA produced by fatty acid β-oxidation in liver mitochondria exceeds the metabolic capacity of the tricarboxylic acid (TCA) cycle, this substance is shunted to the ketone body synthesis pathway (111). Since the liver lacks the key enzymes required for ketone body catabolism, the produced ketone bodies cross the cell membrane via passive diffusion or monocarboxylate transporters 1/2 (MCT1/2), enter peripheral tissues, and serve as the core substrate for subsequent energy decomposition and utilization (112, 113).

Numerous studies have demonstrated that under conditions of limited glucose supply (e.g., prolonged fasting or starvation), ketone bodies can act as an efficient energy source for the brain (31). Under such circumstances, insufficient glucose delivery directly restricts cerebral glucose utilization, while ketone bodies are capable of meeting nearly half of the brain’s basal energy demands, functioning as an indispensable alternative energy substrate for maintaining neural activity (30, 114). Their core advantage lies not only in providing stable energy but also in effectively mitigating excitotoxicity—an effect crucial for protecting neural function and reducing neuronal damage, which can significantly alleviate the adverse impacts of metabolic stress on the nervous system (115, 116).

In addition to serving as essential energy substrates, ketone bodies, particularly β-hydroxybutyrate (β-HB), function as pivotal signaling molecules that regulate adaptive metabolic remodeling in hibernating mammals. Acting as a class I histone deacetylase (HDAC) inhibitor, β-HB epigenetically upregulates the transcription of antioxidant and mitochondrial protective genes, thereby maintaining redox homeostasis and enhancing tissue tolerance during torpor-arousal cycles (117). Furthermore, β-HB acts as an endogenous ligand for GPR109A, which suppresses excessive lipolysis, modulates sympathetic nerve activity, and alleviates inflammatory responses to support systemic energy balance (118). β-HB also serves as a substrate for lysine β-hydroxybutyrylation (Kbhb), a post-translational modification that preserves mitochondrial structure, facilitates mitophagy, and stabilizes metabolic enzyme activity under conditions of hypothermia and hypometabolism (119). Collectively, ketone bodies exert dual roles as both core energy fuels and central regulatory metabolites, thereby promoting cold adaptation, mitochondrial stability, and tissue protection throughout hibernation.

The pathogenesis of hereditary HMG-CoA lyase deficiency fully confirms the central role of ketone bodies in cerebral metabolism. HMG-CoA lyase is the key enzyme mediating the conversion of fatty acid β-oxidation to ketone body synthesis (120). Patients with this disease suffer from impaired ketone body production due to enzyme dysfunction, resulting in hypoglycemia and failure to synthesize ketone bodies during fasting. In the absence of ketone bodies to compensate for insufficient glucose supply, the brain faces energy depletion during fasting, which may lead to permanent encephalopathy and epileptic seizures (121).

**Figure 1:**
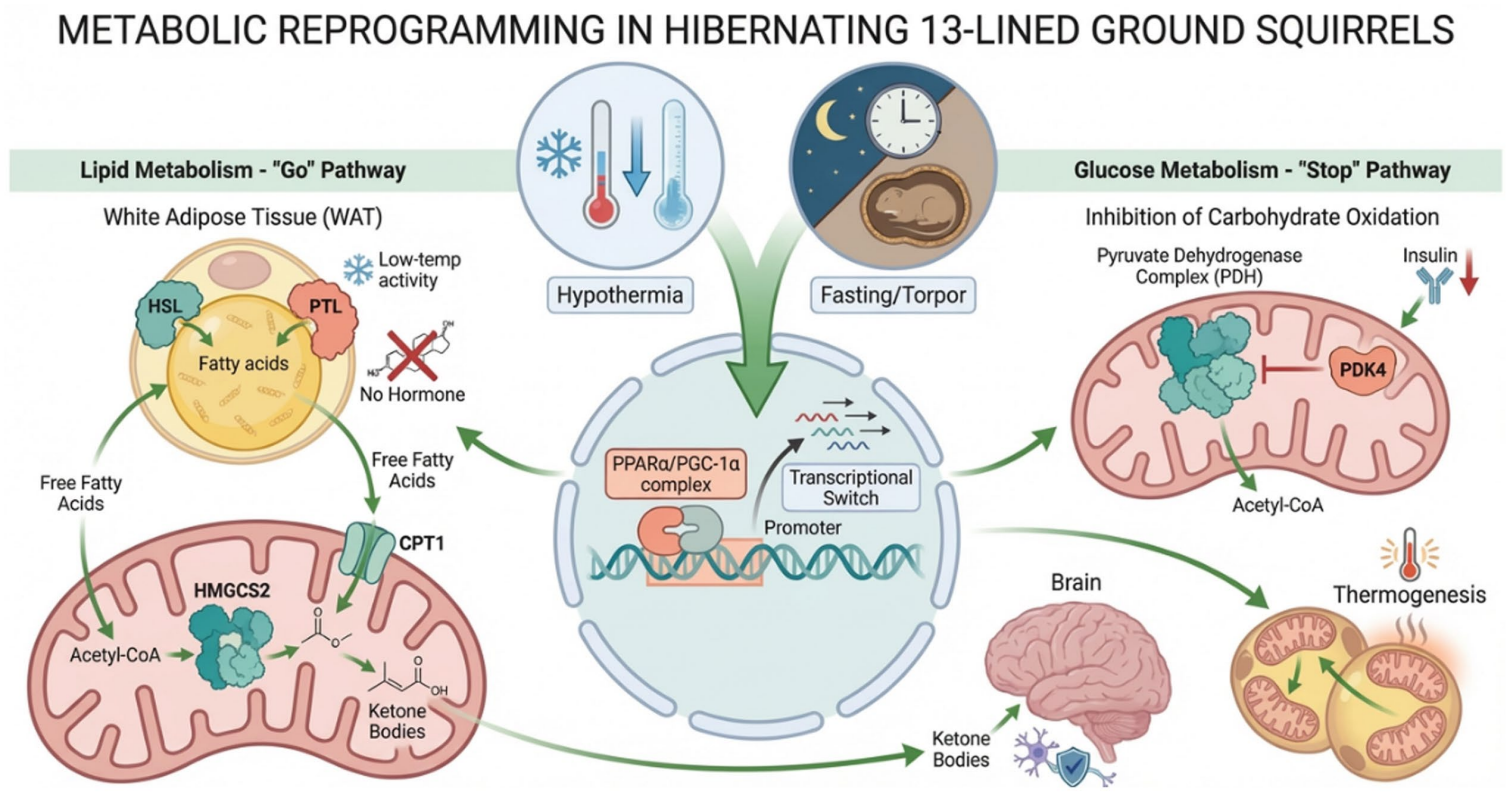
Hypothermia and prolonged fasting/torpor jointly trigger a transcription switch centered on PPARα/PGC-1α. PPARα forms a complex with coactivator PGC-1α, binds to PPRE in the promoter of target genes, and synergistically initiates lipid mobilization and oxidative programs. The left “Lipid Metabolism–Go” pathway shows: WAT undergoes lipolysis under hypothermia and decreased hormone levels; HSL and ectopically expressed PTL with hypothermia- and hormone-independent activity constitute a “dual lipolytic system”, continuously releasing FFA into the blood; FFA enters mitochondria via CPT1 for β-oxidation, and ketone bodies are generated in the liver mediated by HMGCS2, which are transported as key alternative fuels to vital organs such as the brain; meanwhile, part of lipid substrates is allocated to BAT to support thermogenesis. The right “Glucose Metabolism–Stop” pathway shows: PPARα activation further induces PDK4 expression; PDK4 inhibits the activity of PDH, blocks glycolytic products from entering the TCA cycle, thereby suppressing carbohydrate oxidation and enhancing dependence on lipids/ketone bodies; decreased insulin levels and insulin resistance jointly promote this “glucose metabolism brake” effect.

Previous investigations have revealed the association between ketone body synthesis and adaptive thermogenesis in organisms (122). As the key rate-limiting enzyme for ketone body synthesis in liver mitochondria, silencing of HMGCS2 significantly impairs adaptive thermogenesis in mice (123): in the experiment, mice with suppressed ketone body production exhibited marked impairment in the browning of adipose tissues (including epididymal and inguinal fat) after exposure to a low temperature of 12 °C, ultimately leading to decreased thermogenic capacity (124). This finding expands the boundary of the physiological functions of ketone bodies and reveals their multiple roles in regulating energy metabolism. (Figure 1).

#### 3.2.4 Regulation of lipid utilization by skeletal muscle Ca^2+^ futile cycling

In addition to systemic metabolic switches governed by central signaling pathways, skeletal muscle serves as a critical peripheral regulator of lipid homeostasis during hibernation via Ca^2+^ futile cycling—a non-shivering thermogenic mechanism mediated by the ryanodine receptor (RyR), sarcoplasmic reticulum Ca^2+^-ATPase (SERCA) pump, and sarcolipin (SLN). This pathway allows ATP hydrolysis to occur without generating mechanical work, thereby modulating energy expenditure and enhancing fatty acid oxidation in myocytes. As such, it provides a tissue-specific mechanism that complements whole-body lipid metabolic remodeling in hibernators (125–127).

The core molecular machinery of skeletal muscle Ca^2+^ futile cycling consists of three functional components: RyR mediates Ca^2+^ leakage from the sarcoplasmic reticulum (SR) into the cytoplasm; SERCA drives ATP-dependent Ca^2+^ reuptake into the SR; and SLN acts as a key uncoupling peptide that binds SERCA to dissociate Ca^2+^ transport from ATP hydrolysis, enabling heat production without contractile activity. Unlike non-hibernating mammals, where this pathway primarily responds to cold stress, hibernating mammals (e.g., thirteen-lined ground squirrels, Arctic ground squirrels) dynamically regulate this cycle across torpor-arousal cycles to match lipid supply and energy demand.

During deep torpor, hibernators actively downregulate the Ca^2+^ futile cycle to suppress metabolic heat production and conserve lipid reserves. Proteomic and transcriptomic analyses reveal that SLN expression in skeletal muscle (especially the diaphragm, a constitutively active respiratory muscle) is reduced by 5–10 fold during torpor compared to the active state; concurrently, SERCA1a/2a expression is also depressed, minimizing ATP consumption for unproductive Ca^2+^ cycling. This adaptive suppression eliminates non-essential energy expenditure, redirecting stored fatty acids toward vital organ support rather than thermogenesis, and aligns with the global metabolic shift toward lipid sparing during prolonged hypometabolism.

Upon periodic arousal, the Ca^2+^ futile cycle is rapidly reactivated to support thermogenesis and metabolic restart. SLN and SERCA expression rebound significantly, restoring the uncoupled Ca^2+^ cycling that elevates local ATP consumption. The increased ATP demand triggered by this cycle directly enhances fatty acid β-oxidation in skeletal muscle mitochondria, shifting substrate utilization toward intramuscular triglycerides and improving lipid catabolism efficiency. This mechanism not only generates heat to facilitate rewarming but also optimizes lipid turnover, preventing excessive depletion of systemic fat stores while sparing glucose for neural and cardiac function (127).

The regulatory pattern of Ca^2+^ futile cycling exhibits muscle fiber-type specificity in hibernators. Fast-twitch extensor digitorum longus (EDL) muscle shows undetectable SLN expression throughout hibernation, while mixed-fiber-type diaphragm muscle retains dynamic SLN regulation—reflecting a division of labor where respiratory muscle prioritizes lipid-fueled Ca^2+^ cycling for sustained function, while locomotor muscle conserves energy. This tissue-specific adaptation ensures that lipid utilization is precisely allocated to essential functions, avoiding wasteful energy loss during torpor (128).

The RyR/SERCA/SLN-mediated Ca^2+^ futile cycle acts as a peripheral metabolic valve that modulates skeletal muscle lipid utilization efficiency in hibernating animals. By dynamically adjusting the intensity of uncoupled Ca^2+^ cycling across torpor-arousal bouts, hibernators balance thermogenic demand, energy conservation, and lipid homeostasis. This tissue-specific regulatory mechanism expands the flexibility of the global lipid metabolic program, reinforcing the adaptive strategies that enable survival under prolonged cold and food deprivation (127).

### 3.3 Cross-species variations in lipid mobilization patterns

While foundational studies in ground squirrel models have uncovered core principles of lipid metabolism during hibernation, these observations are not universally applicable across the diverse spectrum of hibernating and torpid taxa. Hibernation strategies vary dramatically, from deep multi-day torpor in rodents to shallow seasonal hibernation in bears and short daily torpor in bats and birds, each associated with distinct adaptations in lipid storage, mobilization, and utilization (2). A systematic comparison of typical lipid metabolic traits among mainstream hibernating and torpid species is presented (Table 1). This analysis integrates adipose tissue characteristics (WAT/BAT profiles), species-specific lipid mobilization patterns, and skeletal muscle Ca^2+^ futile cycling regulation, providing a framework to contextualize findings from rodent models within a broader evolutionary and physiological landscape.

**Table 1:** Conserved and species-specific lipid metabolic adaptations in hibernating and torpid species.

Taxonomic group (representative species)	Adipose tissue characteristics (WAT/BAT)	Lipid metabolic features	Skeletal muscle Ca^2+^ futile cycling characteristics
Ground squirrels (*Ictidomys tridecemlineatus*, *Urocitellus parryii*)	Abundant WAT stores; highly developed BAT with active thermogenic function	Preferential mobilization of saturated fatty acids, with retention of linoleic acid and other unsaturated fatty acids	SLN/SERCA/RyR pathway shows cyclic regulation (down in torpor, up in arousal), specific to the diaphragm (126, 127)
Dormice (*Glis glis*, *Eliomys quercinus*)	Distinct WAT lipid accumulation; active BAT with moderate browning	Preferential release of fatty acids based on their oxidizability, with a tendency to retain specific unsaturated fatty acids during certain phases	Stable SLN expression with no significant cyclic fluctuations; Ca^2+^ cycling activity remains at basal levels
Hamsters (*Mesocricetus auratus*, *Phodopus sungorus*)	Abundant WAT stores with inguinal white adipose tissue (iWAT) browning; BAT is present but small in size	Weak selective fatty acid mobilization, with no obvious preference for saturated fatty acid utilization	Constant SLN expression with no cyclic regulation; low Ca^2+^ cycling activity
Bats (*Eptesicus fuscus*, *Rhinolophus ferrumequinum*)	Moderate WAT lipid stores; highly developed BAT with potent non-shivering thermogenesis (NST) function	Body fat is rich in unsaturated fatty acids to support low-temperature oxidation and membrane function; fatty acid mobilization prioritizes substrates based on metabolic availability	Low SLN expression; weak Ca^2+^ cycling activity; thermogenesis relies primarily on BAT-mediated NST
Bears (*Ursus americanus*, *Ursus arctos*)	Abundant WAT lipid stores; developed BAT with low thermogenic demand and no obvious browning enhancement	Fatty acid mobilization favors easily oxidizable substrates with no saturated/unsaturated preference; omega-3 PUFAs are retained in adipose tissue, while short-chain fatty acids are preferentially released	Stable SLN/SERCA expression with no cyclic fluctuations; low Ca^2+^ cycling activity with no apparent thermoregulatory role
Marsupials (*Vombatus ursinus*, *Monodelphis domestica*)	Moderate WAT lipid stores; limited BAT activity with no canonical UCP1 overexpression	Lipid metabolism involves a global shift from carbohydrate to fat oxidation; no evidence of preferential fatty acid mobilization	High SLN expression; Ca^2+^ futile cycling mediates non-shivering thermogenesis to compensate for the lack of BAT-driven thermogenesis
Torpid birds (*Apus apus*, *Archilochus colubris*)	Limited WAT lipid stores; inconspicuous BAT; lipid reserves are primarily intramuscular fat	Short-term torpor is associated with general metabolic depression rather than profound shifts in lipid fuel selection	No SLN expression; SERCA/RyR-mediated Ca^2+^ cycling drives skeletal muscle non-shivering thermogenesis (125)

Corresponding to the interspecific divergence in lipid metabolic strategies, species also differ in their requirement for essential fatty acids, with linoleic acid (C18:2 n-6) serving as a representative example. These differences reflect multiple physiological determinants, the most influential of which is the extent of body temperature decline during hibernation. Deep-hibernating species such as ground squirrels experience extreme hypothermia and therefore require sufficient linoleic acid to maintain cell membrane fluidity, leading to a high reliance on dietary sources (54). In contrast, bears maintain relatively stable body temperatures throughout dormancy, exhibiting lower physiological demand and weaker dependence on exogenous linoleic acid. Additional factors, including the capacity for endogenous synthesis of unsaturated fatty acids, selective retention of fatty acids within adipose tissue, and species-specific overwintering strategies, further shape interspecific variation in linoleic acid utilization and dietary reliance (2, 129).

## 4 Oxidative stress: a critical physiological challenge for hibernating mammal

### 4.1 Main sources of oxidative stress during hibernation

To withstand extreme cold and energy deprivation, hibernating animals have evolved a lipid oxidation-based energy strategy. This strategy persists throughout the hibernation cycle, yet serves as the primary source of oxidative stress during both deep torpor and periodic arousal phases, driven by distinct physiological states (45).

#### 4.1.1 Deep torpor: sustained stress under hypothermia and hypoxia

During deep hibernation, blood flow, heart rate, oxygen consumption, and mitochondrial respiration plummet dramatically. Arctic ground squirrels reduce oxygen consumption by over 90% during hibernation (130). At this stage, the body relies almost entirely on beta-oxidation of fats for energy. The large quantities of reduced coenzymes (NADH and FADH₂) produced by fatty acid breakdown must enter the mitochondrial electron transport chain (ETC) for oxidative phosphorylation. However, under the suppression of low metabolism and hypothermia, electron leakage frequently occurs at complexes I and III within the ETC. This allows electrons to directly combine with oxygen molecules, continuously generating ROS such as superoxide anion (·O₂^−^) (131). Concurrently, prolonged hypoperfusion subjects tissues to relative ischemia and hypoxia, further altering mitochondrial redox states and potentially exacerbating ROS production. Energy-intensive organs with high mitochondrial density, such as the heart and brain, are particularly vulnerable during this process (45).

#### 4.1.2 Intermittent arousal: oxidative burst under ischemia–reperfusion

Periodic awakening serves as a “metabolic reboot and repair window” essential for the long-term survival of hibernating animals. During this phase, physiological functions such as heart rate, blood flow, and body temperature rapidly recover to normal or even elevated levels within hours, accompanied by a sharp increase in mitochondrial respiration rates (132). Data indicates that squirrels’ oxygen consumption during awakening is three times that of their active summer state and 36 times that of hibernation. Mitochondrial respiration studies reveal that ground squirrel liver respiration surges by 70% during awakening compared to hibernation (133). Theoretically, this abrupt oxygen demand increase accompanies a mitochondrial respiratory burst, causing a dramatic rise in ROS production and resulting in “oxidative shock.” Simultaneously, blood flow restoration to tissues in an “energy-starved” state due to prolonged hypoperfusion triggers a classic ischemia–reperfusion injury process. During this process, hypoxanthine accumulated during ischemia undergoes explosive production of superoxide radicals upon oxygen restoration, catalyzed by xanthine oxidase.

#### 4.1.3 Hibernating animals possess unique antioxidant defense mechanisms

Although the theoretical analysis above suggests that hibernating animals face severe oxidative stress challenges during both dormancy and arousal phases, extensive experimental observations reveal a striking paradox: Compared to their active counterparts in summer, many hibernating animals (such as Arctic ground squirrels) show no detectable significant tissue oxidative damage during hibernation cycles. Direct markers of oxidative damage, such as lipid peroxidation and protein carbonylation, do not significantly increase in vital organs like the liver and brain (134–136). This phenomenon strongly suggests that hibernating animals may have evolved highly efficient, unique antioxidant defense and damage repair mechanisms. This mechanism enables them to mitigate the oxidative risks associated with fat-dependent energy supply, thereby successfully maintaining redox homeostasis and preventing tissue damage throughout the prolonged hibernation period.

Suppression of basal mitochondrial ROS production serves as the primary protective strategy during hibernation, while activation of antioxidant enzymes acts as a secondary, compensatory mechanism. Instead of relying solely on ROS scavenging, hibernating animals minimize oxidative stress risk at the source by maintaining mitochondrial structural stability and optimizing fatty acid oxidation–related respiratory efficiency (45). In other words, by reducing ROS production on the one hand and clearing excess ROS on the other, hibernating animals avoid overt oxidative stress damage despite dramatic metabolic fluctuations across the torpor–arousal cycle. However, species-specific differences exist: ground squirrels undergo repeated torpor-arousal cycles and display pronounced cyclic shifts: ROS suppression dominates during torpor with minimal metabolic activity, while antioxidant systems are robustly activated during arousal to counteract surging ROS production (135). By contrast, bears maintain minimal body temperature fluctuations throughout hibernation and rely more on sustained low ROS generation instead of acute antioxidant responses (137).

Studies have proposed that the Nrf2/Keap1 pathway plays a core regulatory role in the antioxidant stress response of animals. This pathway achieves dynamic and controllable regulation of oxidative stress by modulating the tissue-specific antioxidant enzyme system (138). Under basal conditions, unphosphorylated Nrf2 is sequestered in the cytoplasm by the Keap1 protein. When the organism is exposed to oxidative stress, Nrf2 is released from Keap1 and undergoes phosphorylation to form active p-Nrf2. The study found that during deep hibernation, the level of H₂O₂ in tissues such as the heart and brain increases significantly; excessive ROS activate the pathway, leading to a marked upregulation of p-Nrf2 expression. p-Nrf2 enters the nucleus, binds to the antioxidant response element (ARE), upregulates the gene expression of antioxidant enzymes including SOD1, CAT, and GPx1, and thereby enhances the antioxidant capacity of the organism (139, 140).

Under cold stress, an imbalance between energy supply and demand in the body triggers the activation of AMP-activated protein kinase (AMPK) (141, 142). The activated AMPK promotes mitochondrial fission by phosphorylating Dynamin-related protein 1 (Drp1), which disrupts the balance between mitochondrial fusion and fission, leading to a state of excessive fission and insufficient fusion, and subsequently inducing mitochondrial structural abnormalities (11, 143).

AMPK is a core sensor protein regulating cellular energy homeostasis, and its full activation strictly depends on phosphorylation of the *α* subunit at Thr172. This critical process is primarily mediated by the upstream kinase LKB1 complex: functional LKB1 exists in cells as a constitutively active LKB1–STRAD–MO25 trimer. Although its own activity is not significantly affected by metabolic status, it dynamically regulates the phosphorylation efficiency of AMPK by sensing cellular energy signals. Under low-energy conditions such as energy depletion, starvation, or exercise, intracellular AMP levels rise and specifically bind to the *γ* subunit of AMPK, inducing a conformational change that greatly enhances LKB1’s ability to recognize and phosphorylate Thr172. This leads to efficient activation of the AMPK pathway and initiates compensatory regulation of energy metabolism. In contrast, during hibernation, fatty acid *β*-oxidation is enhanced, producing large amounts of acetyl-CoA, which provides abundant substrates for the tricarboxylic acid cycle and oxidative phosphorylation, resulting in a marked increase in intracellular ATP levels. The elevated ATP/AMP ratio in turn inhibits AMPK activation; at the same time, high ATP levels compete with AMP for binding to the *γ* subunit, further inhibiting sustained AMPK activation. This mechanism adapts to the hibernation state, which is characterized by low energy expenditure and high energy storage (45, 144, 145). Furthermore, fatty acid oxidation products can activate PPARα, which then forms a functional complex with peroxisome proliferator-activated receptor gamma coactivator 1-alpha (PGC-1α). This complex upregulates the expression of fusion-related proteins, including mitofusin 1/2 (Mfn1/2) and optic atrophy 1 (OPA1). Studies demonstrated that, compared with the cold stress-only group, the experimental group fed a fatty acid-rich diet showed significant amelioration of structural abnormalities such as mitochondrial cristae dissolution under electron microscopy, and this phenomenon was closely associated with the upregulated expression of OPA1 (146–148) (Table 2).

**Table 2:** Key proteins regulating energy metabolism reprogramming in hibernating animals.

Key protein	Target/tissue	Core biological function	Expression/functional change during hibernation
UCP1	Mitochondria in BAT and beige adipocytes	Mediates non-shivering thermogenesis; uncouples mitochondrial respiratory chain from oxidative phosphorylation to dissipate energy as heat	Significantly upregulated; serves as a specific marker for BAT thermogenesis
PPARα	Metabolically active tissues: liver, skeletal muscle, BAT, kidney	Regulates lipolysis, fatty acid β-oxidation, and ketogenesis; drives the metabolic switch from carbohydrate to lipid oxidation	Strongly activated by hypothermia and fasting; markedly enhanced transcriptional activity
PGC-1α	Forms a complex with PPARα and targets promoters of lipid metabolism genes	Co-activates PPARα; promotes lipid mobilization and oxidation; maintains mitochondrial homeostasis	Upregulated; forms the core regulatory axis with PPARα
PDK4	Heart, skeletal muscle, WAT; targets pyruvate dehydrogenase (PDH)	Inhibits PDH activity; blocks glycolytic products from entering the TCA cycle; suppresses carbohydrate oxidation	Significantly elevated at both protein and mRNA levels
PTL	Extra-pancreatic tissues: WAT, heart	Hormone-independent, cold-adapted lipase; hydrolyzes triacylglycerols	Ectopically upregulated; remains functional at 0 °C
HSL	Adipocytes in WAT	Hormone-regulated lipase; cooperates with PTL to mediate fat breakdown	Maintains basal activity; forms a dual lipolysis system with PTL
ATGL	Adipocytes in WAT	Initiates triacylglycerol hydrolysis; rate-limiting for lipolysis	Upregulated by PPARα; promotes lipid mobilization
CPT1	Mitochondrial membrane	Translocates long-chain fatty acids into mitochondria; rate-limiting for β-oxidation	Upregulated; accelerates fatty acid oxidation for energy
HMGCS2	Liver mitochondria	Rate-limiting enzyme for ketogenesis; produces ketone bodies for brain and vital organs	Upregulated; sustains energy supply to the brain under glucose limitation
Nrf2	Universal; targets antioxidant genes (SOD1, CAT, GPx1)	Master transcription factor for antioxidant defense; mitigates oxidative stress during torpor–arousal cycles	Phosphorylated and activated (p-Nrf2); translocates to the nucleus to enhance antioxidant capacity
Keap1	Cytoplasm; binds and sequesters Nrf2	Retains Nrf2 in the cytoplasm under basal conditions; represses Nrf2 activity	Inhibitory effect relieved as Nrf2 dissociates
SIRT3	Mitochondria in liver and muscle	Deacetylates ETS Complex II; reversibly inhibits mitochondrial respiration	Upregulated/activated; mediates reversible suppression of mitochondrial respiration
Mfn1/2, OPA1	Mitochondria in various tissues	Promote mitochondrial fusion; preserve mitochondrial structural integrity	Upregulated by PPARα/PGC-1α; protect against mitochondrial damage
Drp1	Mitochondria	Mediates mitochondrial fission; excessive fission under cold stress causes structural abnormalities	Phosphorylation/activation suppressed by lipid metabolism; reduces mitochondrial injury

## 5 Conclusion

Lipid metabolism constitutes a sophisticated and core regulatory network that supports the survival of hibernating organisms under extreme environmental stress and fluctuating energy supply conditions. Distinct from the traditional cognition that lipids merely serve as passive energy storage substances, accumulating evidence indicates that lipids act as crucial active regulators of physiological homeostasis during hibernation, exerting protective and adaptive effects through a set of coordinated and precise molecular mechanisms.

The adaptive regulation of lipid metabolism in hibernators manifests itself mainly in three aspects. Cell membrane structural remodeling is a fundamental cold-adaptation survival mechanism. Hibernators remodel the fatty acid composition of cell membranes by upregulating the proportion of unsaturated fatty acids, which stably maintains membrane fluidity under low-temperature stress. This structural adaptation further guarantees the normal function of membrane-bound proteins and ion channels, laying a foundation for the sustainment of basic physiological activities in hypothermic environments.

The precise reprogramming of systemic lipid metabolism dominates the energy adaptation of hibernation, realizing the efficient transition from carbohydrate metabolism to lipid-dependent energy supply. The PPARα/PGC-1α signaling axis acts as the central regulatory hub for this metabolic switch. It promotes lipolysis in WAT to release free fatty acids, upregulates the expression of genes related to mitochondrial fatty acid β-oxidation, and accelerates hepatic ketogenesis to provide sustainable energy for vital organs. In coordination with this axis, PPARα-induced PDK4 inhibits the activity of PDH, blocks glycolytic metabolites from entering the tricarboxylic acid cycle, and ultimately suppresses carbohydrate oxidation, further strengthening lipid-dependent metabolic patterns.

A unique dual lipolysis system composed of PTL and HSL ensures continuous fat breakdown under low-temperature conditions. Different from HSL, PTL exhibits hormone independence and low-temperature tolerance, which compensates for the insufficient lipolytic capacity of conventional pathways in hypothermic states and guarantees stable energy supply during long-term hibernation. In addition, regulatory factors including SIRT3 and PPAR families maintain mitochondrial metabolic efficiency during hibernation-induced metabolic suppression. Ketone bodies, as both alternative energy substrates and key signaling molecules, further enhance metabolic flexibility, support thermogenic adaptation, and protect organ functions, enabling hibernators to survive prolonged energy deficiency.

Lipid-mediated antioxidant defense systems effectively alleviate stress damage during hibernation. The frequent physiological transitions between torpor and arousal produce massive reactive oxygen species and severe oxidative stress. The coordinated lipid regulatory network activates endogenous antioxidant defenses, eliminates oxidative damage to cells and tissues, and avoids fatal injury caused by stress responses, which is essential for the long-term survival of hibernators.

Despite the current progress, the molecular regulatory mechanisms underlying lipid metabolism remodeling and energy homeostasis during mammalian hibernation remain incompletely elucidated. Existing studies are mostly limited to phenotypic observations of single tissues, while the inter-organ crosstalk mechanisms and the precise molecular regulatory networks governing metabolic reprogramming are still unclear, restricting the in-depth understanding of hibernation adaptation principles.

Future research ought to focus mainly on exploring the core molecular mechanisms governing lipid metabolism and ketone body circulation throughout hibernation cycles, clarifying the functional roles of pivotal signaling molecules in metabolic transition. Moreover, multi-omics strategies can be adopted to screen key adaptive genes and regulatory targets, revealing the intrinsic mechanisms of physiological stability maintenance under hypothermia and hypometabolism. Meanwhile, exploring the translational potential of hibernation-related metabolic regulation strategies will also greatly promote the in-depth development and practical application of research in this field. In conclusion, deciphering the intricate lipid-mediated regulatory networks of hibernation not only deepens the fundamental understanding of organism adaptive biology, but also provides novel theoretical insights for the clinical treatment of human metabolic disorders, organ preservation, and cold-induced injury repair.

## Glossary

BAT: brown adipose tissue
NST: non-shivering thermogenesis
SFAs: saturated fatty acids
PUFAs: polyunsaturated fatty acids
ROS: generate reactive oxygen species
WAT: white adipose tissue
iWAT: inguinal white adipose tissue
UCP1: uncoupling protein 1
PPAR*γ*: Peroxisome proliferator-activated receptor γ
LCFAs: long-chain fatty acids
FATPs: fatty acid transport proteins
MPA: medial preoptic area
ETS: electron transport system
RQ: respiratory quotient
PPAR*α*: Peroxisome proliferator-activated receptor α
PPREs: PPAR response elements
ATGL: adipose triglyceride lipase
HSL: hormone-sensitive lipase
CPT1: carnitine palmitoyltransferase 1
PDK4: pyruvate dehydrogenase kinase 4
PDH: pyruvate dehydrogenase
PTL: pancreatic triacylglycerol lipase
TCA: tricarboxylic acid
ETC: electron transport chain
AMPK: AMP-activated protein kinase
Drp1: Dynamin-related protein 1
PGC-1α: peroxisome proliferator-activated receptor gamma coactivator 1-alpha
Mfn1/2: mitofusin 1/2
OPA1: optic atrophy 1
